# The Effect of Microcrystalline Cellulose–CaHPO_4_ Mixtures in Different Volume Ratios on the Compaction and Structural–Mechanical Properties of Tablets

**DOI:** 10.3390/pharmaceutics16030362

**Published:** 2024-03-05

**Authors:** Valentyn Mohylyuk, Artūrs Paulausks, Oskars Radzins, Liga Lauberte

**Affiliations:** 1 Laboratory of Finished Dosage Forms, Faculty of Pharmacy, Rīga Stradiņš University, LV-1007 Riga, Latvia; arturs.paulausks@rsu.lv (A.P.);; 2 Baltic Biomaterials Centre of Excellence, Headquarters at Riga Technical University, LV-1658 Riga, Latvia; oskars.radzins@rsu.lv; 3 Department of Orthodontics, Institute of Stomatology, Rīga Stradiņš University, LV-1007 Riga, Latvia

**Keywords:** tablets, microcrystalline cellulose, calcium phosphate, Heckel plot, tensile strength, pressure–displacement profile

## Abstract

Using microcrystalline cellulose (MCC) with plastic behaviour and calcium phosphate anhydrous (CaHPO_4_) with brittle behaviour under compaction is very popular in the pharmaceutical industry for achieving desirable structural–mechanical properties of tablet formulations. Thus, mixtures of specific grades of MCC and CaHPO_4_ were tested in volume proportions of 100-0, 75-25, 50-50, 25-75, and 0-100 at a constant weight-by-weight concentration of sodium stearyl fumarate lubricant, utilizing a state-of-the-art benchtop compaction simulator (STYL’One Nano). Tablet formulations were prepared at 100, 150, 250, 350, 450, and 500 MPa, and characterized by tabletability profile, ejection force profile, proportion–tensile strength relationship, proportion–porosity relationship, pressure–displacement, and elastic recovery profiles, as well as by in-/out-of-die Heckel plots and yield pressures. Interestingly, the 25-75 formulation demonstrated a two-stage out-of-die Heckel plot and was additionally investigated with X-ray micro-computed tomography (µCT). By post-processing the µCT data, the degree of brittle CaHPO_4_ particles falling apart, along with the increasing compression pressure, was quantified by means of the surface area to volume (S/V) ratio. For the 25-75 formulation, the first stage (up to 150 MPa) and second stage (above the 150 MPa) of the out-of-die Heckel plot could be attributed to predominant MCC and CaHPO_4_ deformation, respectively.

## 1. Introduction

Tablet formulations are one of the most efficient ways of producing pharmaceutical and nutraceutical products. The blending of two or more components with specific properties and proportions enables the production of a tablet with desirable properties [1]. Structural–mechanical properties of tablets are of particular importance. Mechanical properties can influence the possibility and quality of further processing, such as coating and packaging, while structural properties can influence the biopharmaceutical attributes of tablets, such as the dissolution of the drug. Additionally, during the reverse engineering of generic tablet development, one of the most important tasks is the de-formulation of the composition of the original product, as well as the understanding of functions and the behaviour of excipients upon processing.

Mixtures of plastic and brittle deforming compounds can lead to a positive compactibility interaction [2,3,4]. Among the available examples, the combination of plastic microcrystalline cellulose (MCC) and brittle calcium phosphate (CaHPO_4_) is well known and very popular in tablet formulation [4,5]. Both excipients are represented as multiple grades from multiple manufacturers. MCC grades are different in terms of their size, shape, surface area, crystallinity, and moisture content, while CaHPO_4_ can vary regarding its granular form (as aggregated crystals) [6,7,8,9,10,11]. The grades and proportions of MCC and CaHPO_4_ can influence the compression, mechanical properties, and porosity, and thus the water ingress, disintegration, and drug release of tablets [12,13,14,15,16]. Notably, CaHPO_4_ is practically insoluble in water, but soluble in diluted acids [6]. Different proportions and grades with different particle sizes of CaHPO_4_ can influence the drug release in vitro, including cone formation [17,18]. Thus, such pH-dependant solubility can influence in vivo drug release as well. Therefore, the grades and proportions of MCC and CaHPO_4_ should be carefully selected, considering both the mechanical and the biopharmaceutical aspects.

In order to better understand the fundamental properties and mutual influence on the structural–mechanical properties, the single components and binary mixtures of MCC and CaHPO_4_ have been investigated. The addition of 25% (*w*/*w*) of MCC to CaHPO_4_ resulted in an increase in compactibility due to increased densification [2]. A simple relationship was observed with increasing mass fractions of MCC on the mean yield pressure and elastic recovery of the compacts. At all force application rates, the mean yield pressure decreased, while elastic recovery increased with increasing mass fractions of MCC, attributed to the dominating plastic deformation effect of microcrystalline cellulose [5]. The binary mixture of MCC and CaHPO_4_ was investigated to determine the relationship between the solid fraction, compressive stress, and mechanical properties. The plastic deformation was found to be predetermined by the percolating cluster-forming component [19]. Based on the compression phase and dwell time (the area–quotient–index) data, the percolation threshold of CaHPO_4_ in the mixture with MCC was estimated [20].

A properly instrumented tablet press is a technical requirement for performing close investigation of the tableting process and improving the information-based understanding of physical processes, along with their consequences on the resulting structural–mechanical properties of the tablets. Recently, equipment such as compaction simulators containing high-tech sensors and sophisticated user-friendly software have become more available [21]. This have facilitated reinvestigations of well-known combinations of excipients such as MCC and CaHPO_4_, as well as detailed considerations.

The aim of this study was to investigate the tableting behaviour of binary mixtures of microcrystalline cellulose and calcium phosphate dibasic anhydrous (represented by CEOLUS^TM^ UF-711 and DI-CAFOS^®^ A60) in different volume ratios at a range of compaction forces in order to observe and explain the influence of mixture composition on the structural–mechanical properties.

## 2. Materials and Methods

### 2.1. Materials

**Figure 1:**
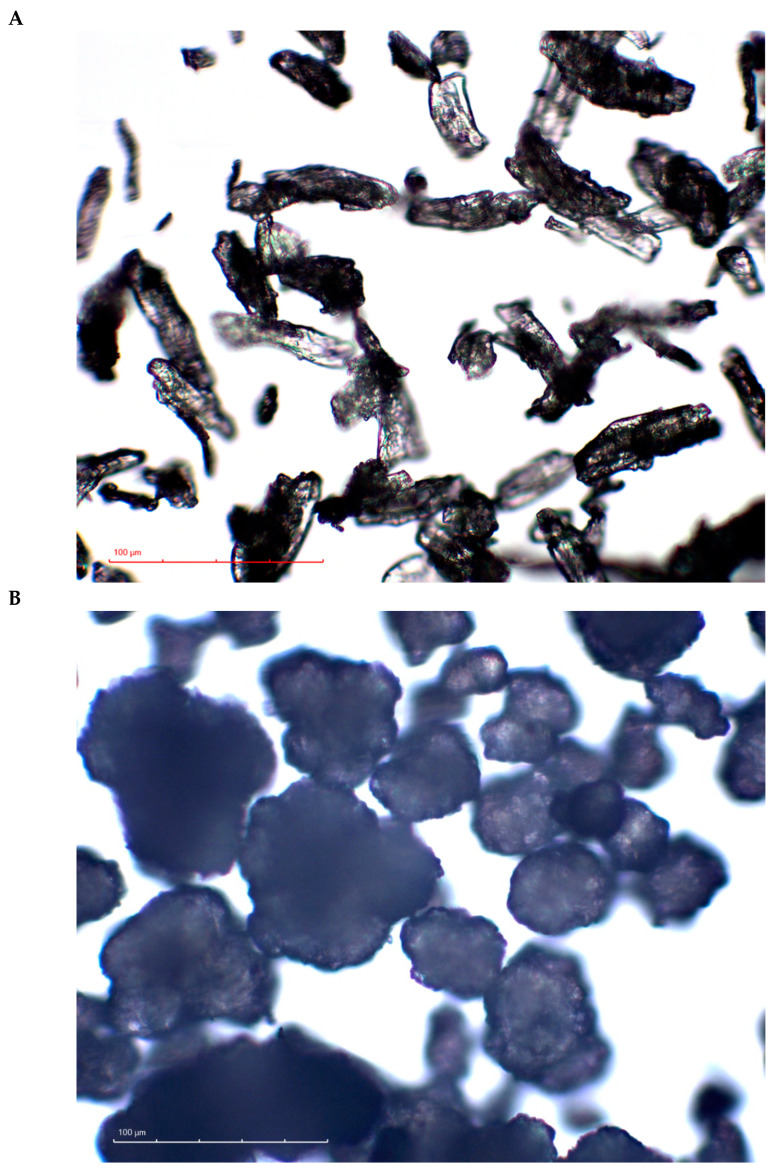
Optical microscopy images of CEOLUS^TM^ UF-711 (**A**) and DI-CAFOS^®^ A60 (**B**) at magnification ×40.

Microcrystalline cellulose (MCC) powder (CEOLUS^TM^ UF-711; Asahi Kasei, Tokyo, Japan; Figure 1A) and calcium phosphate dibasic anhydrous (CaHPO_4_; DI-CAFOS^®^ A60; Budenheim KG, Budenheim, Germany; Figure 1B) were used as primary components for the binary mixtures, while silica (SYLOID^®^ 244FP; Grace GmbH, Worms, Germany) and sodium stearyl fumarate (PRUV^®^; JRS Pharma, Rosenberg, Germany) were introduced into the composition as a glidant and a lubricant, respectively. The specific grades of MCC, CaHPO_4_, silica, and sodium stearyl fumarate were used because of their availability in our laboratory during the planning of the experiment.

### 2.2. Microscopy

CEOLUS^TM^ UF-711 and DI-CAFOS^®^ A60 were observed with an optical microscope (BA410E; Motic, Xiamen, China). The microscope was equipped with a 50 W halogen lamp and Motic EC-H Plan 4×/0.1, 10×/0.25 and 40×/0.65 objective lenses. The images were collected by a MoticamProS5 Lite camera controlled by Motic Images Plus 3.0 software.

### 2.3. Preparation of Powder Mixtures

The volume ratio was calculated based on the true density of components; formulation names reflected the MCC–CaHPO_4_ volume ratio. Powder samples (Table 1) were prepared in accordance with the same multistep procedure. MCC, CaHPO_4_, and silica were mixed for 10 min in a double-cone blender (DVC Developer; Comasa, Barcelona, Spain). To ensure microscopic homogeneity, the obtained mixture was then gently sieved through a 1.0 mm mesh size sieve and mixed again for 5 min. In the next step, sodium stearyl fumarate was sieved (through a 0.5 mm mesh size sieve) and then added to the premix, which then was mixed for 2 min. The obtained mixture was sieved through a 1.0 mm mesh size sieve and mixed again for 2 min.

### 2.4. Preparation of Tablets

Powder mixtures were tableted with 11.28 mm flat punches to obtain a target mass of 500 mg using a STYL’One Nano (Medelpharm, Beynost, France) compaction simulator. Compression cycles simulated a small rotary press with a turret diameter of 180 mm, precompression roll diameter of 44 mm, angle between rollers of 65 degrees, compression roll diameter of 160 mm, angle between main compression and beginning of compression ramp of 60 degrees, angle of ejection ramp of 20 degrees at a simulated tableting speed of 70 rpm (maximum for STYL’One Nano), pre-compaction force of 5 kN (50 MPa), and compaction force of 10–50 kN (100–500 MPa). Powder-feeding into the die was performed automatically via the feed shoe [22].

### 2.5. Tablet Hardness Measurement and Tensile Strength Calculation

The tablet height (*t*), diameter (*d*), and tablet crushing strength (hardness or breaking force, *F*) were measured (*n* = 10) using a tablet tester (ST50 WTDH; SOTAX AG, Aesch, Switzerland) immediately after compaction. The radial tensile strength (*τ*, MPa) was calculated by employing the following equation [23]:

(1) $$\tau =\frac{2\;F}{\pi \;d\;t}$$

### 2.6. Calculated True Density

The calculated true density of the tablet composition was obtained based on the pycnometric density (*ρ_t_*) of MCC (1.586 g/cm^3^) [7,24], calcium phosphate dibasic anhydrous (2.890 g/cm^3^) [6], sodium stearyl fumarate (1.110 g/cm^3^) [25], silica (2.200 g/cm^3^) [26], and their shares (*x*, *w*/*w*), using the additive methodology and the following equation [27]:

$$\rho_{t}=\left(\rho_{exc\;1}\times x_{exc\;1}\right)+\left(\rho_{exc\;2}\times x_{exc\;2}\right)+\cdots +\left(\rho_{exc\;i}\times x_{exc\;i}\right)$$

### 2.7. Apparent Density, Porosity, and Solid Fraction Calculation

The relative volumes and densities of the tablets were determined after ejection from the die. The apparent density (*ρ_a_*) of the tablets was calculated as the ratio of tablet weight (*w_tab_*) and volume of cylinder using the following equation:

(2) $$\rho_{a}=\frac{w_{tab}}{\pi {\left(\frac{d}{2}\right)}^{2}t}$$

The solid fraction (*SF*) and porosity (*ε*) of the tablet was calculated using the following equations [28]:

(3) $$SF=\frac{\rho_{a}}{\rho_{t}}=(1-\varepsilon )$$

(4) $$\varepsilon =1-\frac{\rho_{a}}{\rho_{t}}=(1-SF)$$

### 2.8. Heckel Plot Construction

The in-die Heckel plot—the relative density *ln*(1/*ε*)—was calculated using Alix software version 20220711 (Medelpharm/Korsch, Berlin, Germany) [29]. The relative density and compaction pressure (*P*, MPa) data were plotted in accordance with the Heckel equation [30]:

(5) $$\ln\left(1/\varepsilon \right)=K\times P+\ln\left(1/\varepsilon_{0}\right)=K\times P+A$$

where *K* is the slope of the linear region (the proportionality constant) and *ln*(1/*ε*_0_) is a constant, *A*, which represents the degree of packing (at porosity *ε*_0_) achieved at low pressure because of the rearrangement process before an appreciable amount of interparticle bonding takes place. For the out-of-die Heckel plot, the relative density *ln*(1/*ε*) was calculated based on the ejected tablet volume and calculated porosity (*ε*) for every compaction pressure (*n* = 10). For all formulations, the linear region was chosen as between 100 and 150 MPa, while for F 25-75 and F 0-100, the linear region between 250 and 450 MPa was analysed. *Py* was calculated in accordance with Hersey and Rees, using the following equation [31]:

(6) $$P_{y}=\frac{1}{K}$$

### 2.9. Elastic Recovery

The elastic recovery (*ER*) was computed as a percentage of tablet recovery based on the tablet height after ejection (*h_ejected_*) and the in-die minimum thickness (*h_in-die_*) [29], using the following equation:

(7) $$ER,\%=\frac{h_{ejected}-h_{in-die}}{h_{in-die}}\times 100\%$$

### 2.10. X-ray Micro-Computed Tomography (µCT) of Tablet Samples

A small piece of the tablet (approximately 5 mm^3^) was broken off and scanned using a 3D Micro X-ray CT Scanner (CT Lab HX; Rigaku Corp., Tokyo, Japan) at 70 kV with a current of 50 µA, focus set on S, and with no filter. Scan acquisition settings were 5 FOV, long geometry, high-resolution scan mode, and super high image resolution (68 min scan time). Tablet CT scan images were exported as DCM files and processed with dragonfly software (Dragonfly version 2022.2.0.1409; Object Research Systems (ORS) Inc., Montreal, QC, Canada). From these scans, a representative 3D-section of tablet sample internal volume was isolated using the “masking with cylinder” tool. Briefly, a cylindrical piece of the 3D image inside tablet volume was isolated from the whole 3D image. The image piece was then split into three regions of interest (ROI) by selecting regions in the voxel intensity histogram. First, the CaHPO_4_ particles (because of the high contrast); second the non-CaHPO_4_ space, which consisted of MCC, other excipients, and air; and third, the whole space (first and second). The volume percentage of CaHPO_4_ particles and the non-CaHPO_4_ space was calculated against the whole space ROI. The surface area of CaHPO_4_ particles was calculated using the Lorensen algorithm and normalized for each sample by dividing by the volume of the masking cylinder. Based on the CT method used, the radiographic projections were combined to construct 2D sections of the tablet sample, followed by the 3D sample reconstruction. The final images were composed of voxels with different grey levels corresponding to different density values [32]. The adjustment of grey levels influenced the determined volume of CaHPO_4_, but was used identically for all processed samples.

## 3. Results and Discussion

**Figure 2:**
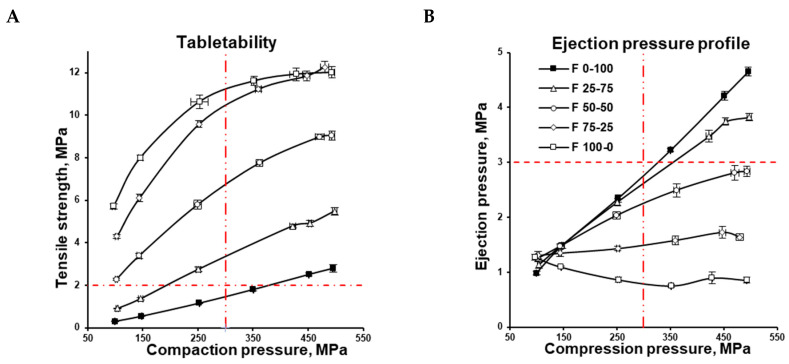
Tablet tensile strength (**A**) and tablet ejection pressure (**B**) as functions of compression pressure.

Deformation upon tableting can surely be considered volumetric deformation, which refers to the dependence of the compression behaviour on the volume ratios of the components. The volume fractions of single components in the binary mixture, along with their mechanical properties, should predetermine the deformation of binary mixtures and result in the mechanical properties of tablets. Under compaction, the volume of tablet constituents approaches its true volume. Therefore, after compaction, the volume of components is relatively close to their true volume. Thus, the effect of the volume ratio of components (calculated based on the pycnometric density of single components) on the deformation upon tableting and mechanical properties of the tablets was of interest, and was used in the formulations to achieve specific MCC and CaHPO_4_ proportions (Table 1). The increase in the CEOLUS^TM^ UF-711 fraction decreased the flowability of the powder mixture; thus, a fixed weight-by-weight concentration of glidant was added to every formulation. At the same time, the weight-by-weight concentration of lubricant sodium stearyl fumarate was kept constant across all formulations. This resulted in the increases in glidant and lubricant volume shares, along with the increase in high true-density CaHPO_4_ proportions. However, the lubricant amount was justified by the tablet ejection force profile (Figure 2B): at compression pressures up to 300 MPa, the ejection pressure was not higher than 3 MPa [33].

The tensile strength of the MCC formulation (F 100-0) increased along with the compression pressure in the range of 100–450 MPa, although increasing the compression pressure from 450 to 500 MPa did not increase the tensile strength further (Figure 2A). At the same time, the CaHPO_4_ formulation (F 0-100) demonstrated an almost linear increase in tablet tensile strength from 100 to 500 MPa. At each compression pressure, the tablet tensile strength of the MCC formulation was significantly higher than the tensile strength of the CaHPO_4_ formulation. The decrease in MCC content in the formulation was accompanied by a decrease in tablet tensile strength (Figure 2A), and was in agreement with the available literature data [5].

Based on the assumption that the hardness of a tablet reflects the bonding that occurs upon tableting [1], the MCC cohesion could be concluded to be higher than MCC–CaHPO_4_ adhesion, which itself is higher than CaHPO_4_ cohesion. Nevertheless, the intermolecular forces could be filtered out by the lubricant film [34,35,36]. Thus, the possible effect of the lubricant on the bonding efficiency cannot be ignored. DI-CAFOS^®^ A60 has a lower apparent specific surface area compared with CEOLUS^TM^ UF-711 (Figure 1), accompanied by a lower specific volume of DI-CAFOS^®^ A60 because of its higher true density (2.890 vs. 1.586 g/cm^3^, respectively; Table 1). This makes it more susceptible to an increase in lubricant content. In other words, in addition to comparably lower CaHPO_4_ cohesion, at a lower specific surface area, the effect of the same amount of lubricant on decreasing the tensile strength is more pronounced. However, even at an increased volume share of lubricant, an increase in CaHPO_4_ volume share and compression force resulted in an increase in the ejection force (Table 1, Figure 2B).

Usually, 300–500 MPa is the highest pressure level for pharmaceutical tableting [37]. For a successful formulation window, 300 MPa is widely considered the maximum allowable compression pressure for shaped tooling; 2 MPa is widely considered the minimum acceptable tensile strength of tablets to comply with both pharmacopeial requirements and downstream processing [33]. The abovementioned maximum allowable compression pressure for shaped tooling and the minimum acceptable tensile strength are depicted as vertical and horizontal red lines, respectively (Figure 2A). Thus, from the practical point of view, at conditions simulating a small rotary press at 70 rpm, the F 0-100 CaHPO_4_ formulation could only yield desirable tablet tensile strength at a compression pressure of 350 MPa, while the F 25-75 mixture demonstrated this at more than 150 MPa. At the same time F 50-50, F 75-25, and F 100-0 could yield a desirable tablet tensile strength even at a relatively low compression pressure of 100 MPa (Figure 2A).

**Figure 3:**
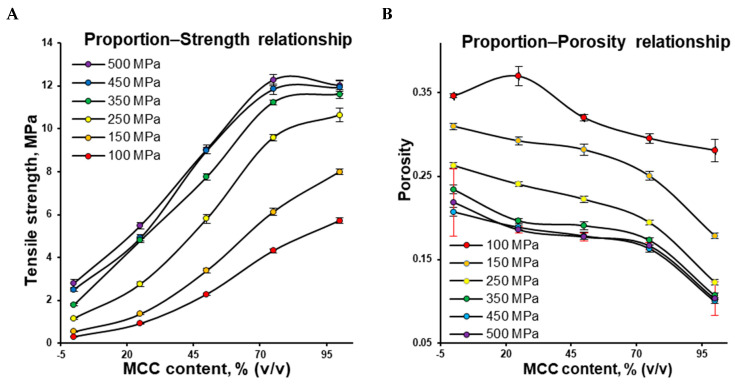
Tensile strength as a function of the MCC content—proportion–tensile strength relationship profiles (**A**) and porosity as a function of the MCC content—proportion–porosity relationship profiles (**B**; for clarity, error bars at 500 MPa are highlighted in red).

Upon the application of compaction pressure, the decrease in porosity increases the contact area, bonding, and tensile strength. However, the ability of ingredients and their mixtures to decrease porosity as a function of pressure is different and can be characterized by the mean yield pressure. Interestingly, the component proportion–tensile strength profiles obtained at all compaction pressures in the range of MCC volume shares from 25% to 75% showed an almost linear relationship (Figure 3A), where the tensile strength increased along with the MCC content and compression pressure. In the same MCC volume share range, the proportion–porosity profiles also demonstrated an almost linear relationship (Figure 3B), where the porosity reduced along with the MCC content and compression pressure. The non-linear relationship in the range of MCC volume shares from 0% to 25% and from 75% to 100% could be attributed to percolation thresholds of CaHPO_4_ and MCC, respectively (Figure 3).

The proportion–porosity relationship (Figure 3B) can be utilized to explain how the tablet’s tensile strength (Figure 3A) is controlled by density. This is because the powder can no longer be compacted further beyond a certain compaction pressure. In contrast to the compression pressure range of 100–350 MPa, a relatively small change in porosity resulted in a relatively small change in tensile strength at 350–500 MPa (Figure 3B vs. Figure 3A).

**Figure 4:**
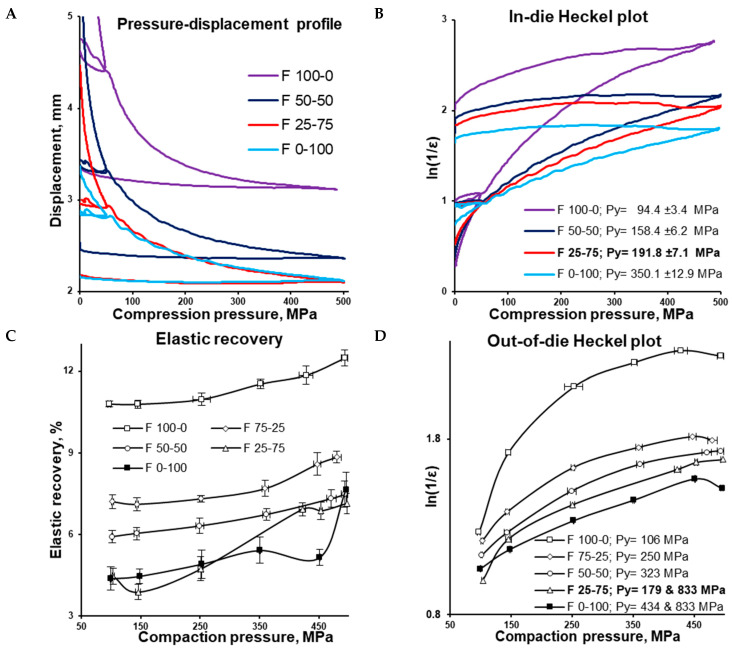
In-die pressure-displacement profile (**A**), in-die Heckel plot for F 100-0, F 50-50, F 25-75, and F 0-100 formulations (**B**), elastic recovery (**C**), out-of-die Heckel plot for F 100-0, F 75-25, F 50-50, F 25-75, and F 0-100 formulations (**D**).

In-die pressure–displacement profiles reflected the difference in the true density of formulations. To apply the same preset compression pressure to CaHPO_4_-rich formulations, the distance between the punches should be smaller, resulting in tablets being thinner because of the higher true CaHPO_4_ density compared with MCC (Figure 4A). In addition to the decreasing MCC portion and the increasing CaHPO_4_ portion, the in-die yield pressure of formulations gradually increased from 94 MPa (F 100-0) to 350 MPa (F 0-100) (Figure 4B). As far as in-die yield pressure can be used as an indicator of material plasticity [38], the plasticity of formulations expectably increased from the CaHPO_4_ formulation to the MCC formulation (from F 0-100 to F 100-0, respectively). Considering the measured values for formulations with MCC–CaHPO_4_ combinations, their in-die yield pressure cannot be predicted by additive methodology (such as the calculated true density) based on the in-die yield pressures of F 0-100 and F 100-0.

As expected, after ejection from the die, the increase in the MCC portion increased the value of elastic recovery (Figure 4C). F 100-0, F 75-25, and F 50-50 demonstrated increases in elastic recovery values with the increase in compaction pressure. The elastic recovery profile of F 0-100 had relatively large standard deviations, while F 25-75 had a comparably complex shape, and the elastic recovery of F 25-75 and F 0-100 also increased along with the compaction pressure.

**Figure 5:**
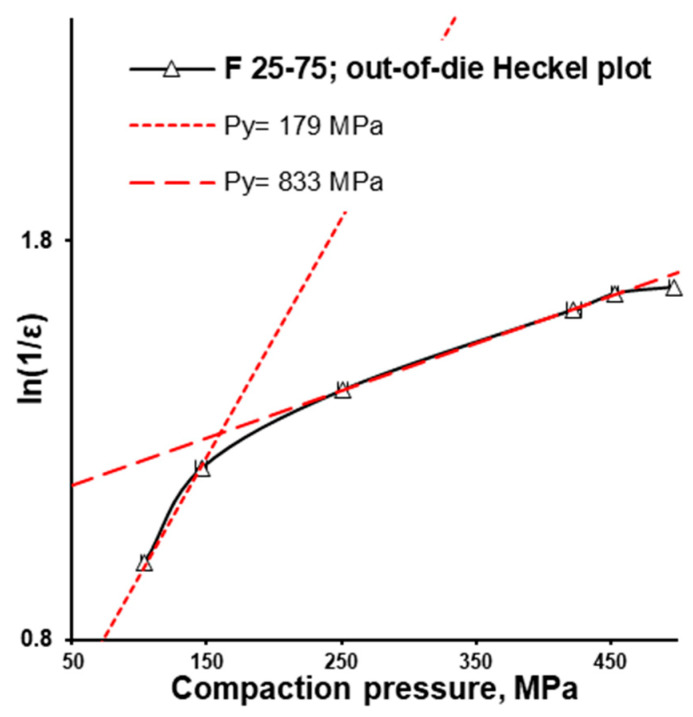
F 25-75 formulation: out-of-die Heckel plot.

The difference between in-die and out-of-die tablet densities depends on elastic recovery. In accordance with the out-of-die Heckel plot (and in agreement with the in-die Heckel plot), the out-of-die yield pressure of the MCC formulation (F 100-0) was the smallest, equalling 106 MPa, while the out-of-die yield pressure of the CaHPO_4_ formulation (F 0-100) was the highest, equalling 434 MPa (Figure 4D). Interestingly, being surrounded with F 50-50 (Py = 323 MPa) and F 0-100 (Py = 434 MPa), the F 25-75 (Py = 179 MPa) formulation did not follow the sequence of the out-of-die yield pressure increases along with the CaHPO_4_ proportion increase. However, the angle of the first part of the curve was close to that of the MCC formulation (F 100-0), while the rest of the curve was parallel to the CaHPO_4_ formulation (F 0-100). This similarity was supported by the same Py (833 MPa) for the compared 250–450 MPa compression pressure range (Figure 4D). Thus, it can be proposed that the densification of F 25-75 was predetermined by MCC (despite the low MCC portion) in the compaction pressure range of 100–150 MPa, and dictated by CaHPO_4_ (Figure 5) in the compaction pressure range of 150–500 MPa.

**Figure 6:**
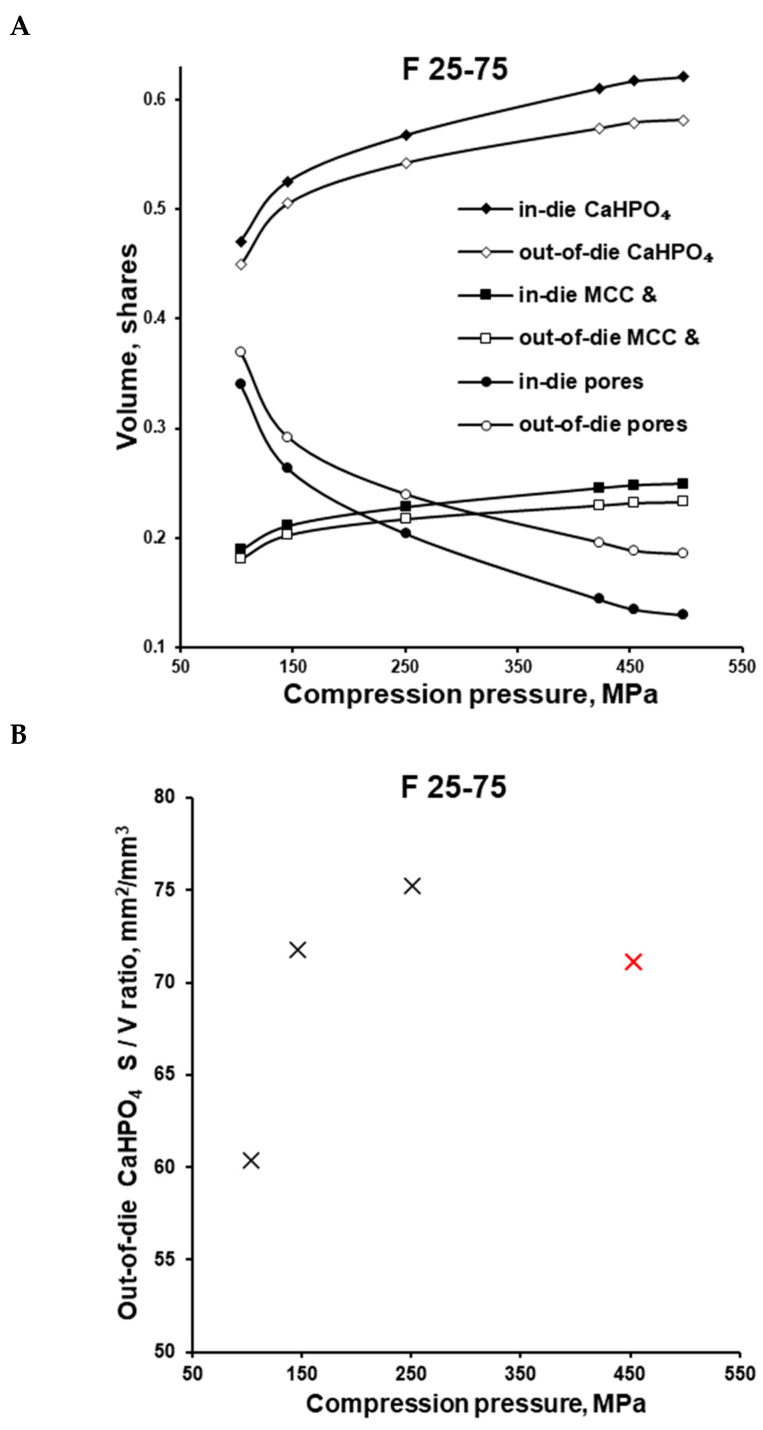
F 25-75 formulation: calculated volume shares of tablet constituents at different compression pressures based on the in-die and out-of-die tablet dimensions (**A**); out-of-die surface/volume ratio of tablets prepared at different compression pressures based on the µCT measurements (**B**).

**Figure 7:**
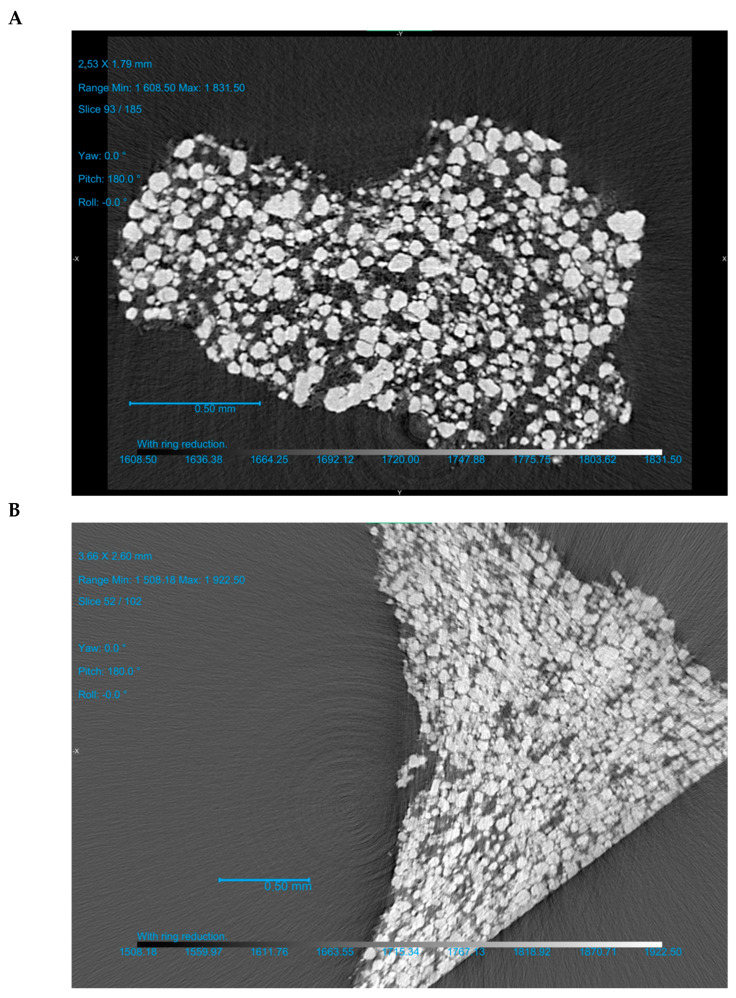
F 25-75 formulation: µCT scan of the piece of the tablet prepared at compression pressures of 104 MPa (**A**) and 454 MPa (**B**). Three-dimensional reconstruction videos of F 25-75 based on the µCT measurements are available in the Supplementary Materials.

The F 25-75 formulation was investigated more thoroughly. After the ejection of the tablet (out-of-die vs. in-die), the volume share of components in the apparent tablet volume decreased due to the elastic recovery. At 100, 150, and 250 MPa and even higher compaction pressures, the calculated volume share of CaHPO_4_ comprised 0.471, 0.526, and 0.568, and higher in-die values, as well as 0.450, 0.505, and 0.542, and higher out-of-die values, respectively (Figure 6A, Table 2). The calculated CaHPO_4_ volume share increased along with the increase in compression pressure, and the same trend was observed for the µCT measured CaHPO_4_ volume share (Figure 6A vs. Figure 6B). Despite the MCC-like out-of-die Heckel plot profile in the compaction pressure range of 100–150 MPa, the µCT investigation showed the increase in surface area to volume (S/V) ratio (100 vs. 150 MPa; Figure 6B), witnessing the fracture of CaHPO_4_ particles along with the increase in compaction pressure from 100 to 150 MPa. The subsequent compaction pressure of 250 MPa further increased the CaHPO_4_ S/V ratio, witnessing the further fracturing/brittle deformation of CaHPO_4_ particles (Figure 6B). Nevertheless, despite the decreases in the logical sequence of the CaHPO_4_ volume share and the visual CaHPO_4_ particle size (Figure 7, Supplementary Materials), the CaHPO_4_ S/V ratio value at 450 MPa unexpectedly turned out to be too low (Figure 6B). The authors considered the S/V ratio of CaHPO_4_ at 450 MPa to be wrong and predetermined with the limitations of the µCT settings.

## 4. Conclusions

Formulations of MCC–CaHPO_4_ with volume share proportions of 100-0, 75-25, 50-50, 25-75, and 0-100, and their respective lubricant volume shares of 2.8, 3.4, 4.0, 4.5, and 5.0 were investigated. The simultaneous increases in both the lubricant volume share and the CaHPO_4_ volume share were justified by the ejection force profile. Next to the decrease in the MCC volume share, the decrease in the tablet tensile strength was observed, which can be partially explained by the increase in the lubricant volume share. For all formulations, upon the increase in compression pressure, decreases in porosity and increases in tensile strength were shown. At an MCC volume share range of 25% to 75%, the increase in tensile strength and the decrease in porosity fraction was observed in a linear manner. The F 25-75 formulation demonstrated a complex elastic recovery profile and two-stage out-of-die Heckel plot. The first stage could be attributed to predominant MCC; the second stage could be attributed to CaHPO_4_ deformation. The level of brittle deformation of CaHPO_4_ in the F 25-75 formulation in the compression force range of 100–250 MPa was additionally illustrated using the µCT method. In this investigation, the compaction simulator was shown to be a powerful tool for the deep and detailed investigation of composition variables, the compaction process, and their effect on the properties of the resulting tablets. The investigation of the combinations of different MCC and CaHPO_4_ grades can be considered a potential direction for future work.

## Figures and Tables

**Table 1:** Mixture and tablet compositions.

Ingredients	True Density	F 100-0	F 75-25	F 50-50	F 25-75	F 0-100	F 100-0	F 75-25	F 50-50	F 25-75	F 0-100
	mg/mm^3^	*w*/*w*					Volume, %				
CEOLUS ^TM^ UF-711	1.586	0.977	0.608	0.346	0.151	0.000	96.9	72.3	47.9	23.8	0.0
DI-CAFOS^®^ A60	2.890	0.000	0.369	0.631	0.826	0.977	0.0	24.1	47.9	71.4	94.6
PRUV^®^	1.110	0.020	0.020	0.020	0.020	0.020	2.8	3.4	4.0	4.5	5.0
SYLOID^®^ 244FP	2.200	0.003	0.003	0.003	0.003	0.003	0.2	0.3	0.3	0.3	0.4
∑	–	1.000	1.000	1.000	1.000	1.000	100.0	100.0	100.0	100.0	100.0
*Calculated* *true density*	–	*1.578*	*2.060*	*2.401*	*2.655*	*2.852*					

**Table 2:** F 25-75 formulation: calculated volume shares of tablet constituents based on the in-die and out-of-die tablet dimensions at different compression pressures.

		Compression Pressure (MPa)					
Volume Shares of		104	146	251	423	454	498
**in-die**	CaHPO₄	0.471	0.526	0.568	0.611	0.617	0.621
	MCC and the other excipients	0.189	0.211	0.228	0.245	0.248	0.249
	porosity	0.340	0.264	0.204	0.144	0.135	0.130
	∑	1.000	1.000	1.000	1.000	1.000	1.000
**out-of-die**	CaHPO₄	0.450	0.505	0.542	0.574	0.579	0.581
	MCC and the other excipients	0.180	0.203	0.218	0.230	0.232	0.233
	porosity	0.370	0.292	0.240	0.196	0.188	0.185
	**∑**	1.000	1.000	1.000	1.000	1.000	1.000

## Data Availability

The data presented in this study are available on request from the corresponding author.

## Supplementary Materials

Three-dimensional reconstruction videos of F 25-75 based on the µCT measurements are available through the following links: sample of tablet compressed at 104 MPa, https://youtu.be/TjBvCZMOk3o (accessed on 26 February 2024); sample of tablet compressed at 146 MPa, https://youtu.be/3bLDvqOYWHM (accessed on 26 February 2024); sample of tablet compressed at 251 MPa, https://youtu.be/TRUuYbWZVIE (accessed on 26 February 2024); sample of tablet compressed at 454 MPa, https://youtu.be/hb_BvkQS4WA (accessed on 26 February 2024).
